# High-Contrast Marking of Stainless-Steel Using Bursts of Femtosecond Laser Pulses

**DOI:** 10.3390/mi14010194

**Published:** 2023-01-12

**Authors:** Simas Butkus, Vytautas Jukna, Evaldas Kažukauskas, Žilvinas Svirksas, Domas Paipulas, Valdas Sirutkaitis

**Affiliations:** Laser Research Center, Vilnius University, Sauletekio Av. 10, LT-10223 Vilnius, Lithuania

**Keywords:** black-marking, white-marking, high-contrast marking, femtosecond burst mode, controlled melting

## Abstract

The marking and surface structuring of various materials is important in various industrial fields such as biomaterials, luxury goods, anti-counterfeiting, automotive and aerospace, electronics and semiconductor industries, and others. Recent advances in laser technology, such as burst-mode lasers, have opened new ways of affecting the surfaces of various materials, inducing a different appearance and/or properties of the laser-exposed areas. From earlier studies, it is known that when splitting a single pulse into multiple pulses and thus creating a quasi-MHz–GHz repetition rate regime, it is possible to increase not only the ablation efficiency but it also provides the possibility to tune the heat in-flow into the surface. Such new regimes enable the control of the surface roughness as well as the optical properties and corrosion resistance. In this work, we analyze the effect of the different burst-mode regimes for the marking of stainless-steel samples, aiming to produce high-contrast marking having different shades of black/white color (black-gray-white). Moreover, we investigate the angular dependence of the reflected light after laser treatment numerically from the measured surface morphology

## 1. Introduction

The laser-induced colorization of materials arouses great interest in the fields of industrial marking, security, and arts [1]. Though non-laser marking methods of various surfaces are still applicable [2], laser marking offers unique advantages (non-contact, durable, micrometer scale accuracy, high speed, high productivity and low operation costs, high degree of automation, etc.), and provides a broad range of possibilities using a single source. The aim of laser marking is to perform a permanent trace on the surface of the material to make it readable and recognizable. Typically, laser marking/engraving is performed via ablation-based methods [3] by changing the surface roughness of the exposed area, thus increasing/reducing the amount of reflected light. Surface melting via CW or long pulse laser systems improves several surface functional properties [4,5,6]. Heat induced oxide layer formation [7,8,9], where the surface of a metallic sample is heated and forms a transparent oxide layer, which is based on the thickness of the layer due to the interference of light, suppresses specific wavelengths. Laser induced periodic surface structure formation (LIPSS) [10,11,12,13,14,15,16] is where the periodic structures act as diffraction gratings and reflect different wavelengths at different angles, thus creating a colored appearance of the exposed areas. Other methods regarding surface marking, such as color appearance due to surface plasmonic resonance [17], chemical composition change induced contrast [18], and randomly distributed nanoparticles [19], have also been reported.

Recently, new advancements in laser technology, such as the burst mode regime, were demonstrated to have far better efficiency in micromachining materials than the conventional systems [20,21,22,23,24,25]. The stated technique utilizes the separation of energy of a single pulse into multiple pulses (burst) by a certain temporal delay. The temporal separation can vary from ~100 ps to ~10 ns. In this case, the relatively high pulse energy can be divided into a packet of pulses with pulse energy values that are closer to the optimal ablation rate; moreover, it can increase or reduce thermal effects in surface ablation [26]. When a train of pulses impinges the surface the properties of the material are altered, i.e., the temporal separation is small enough that the heat does not diffuse away until the following intra-burst pulse arrives, and the heat can start to build up locally in the irradiated region. In this case, given the right conditions, the reflectivity of the metal can decrease from >95% (reflection at room temperature for the first pulse) to <80% (for the trailing pulse) if two pulses are separated by ~100 ps [27]. However, the modelling of the energy absorption and pulse propagation in time and space domains is a complicated task, since one should consider the presence of the remaining plasma. Numerous reports [28,29,30,31] suggest that after a femtosecond pulse hits the surface, the ablated mass, which initially consist of a mixture of hot electrons, ions, and nanoparticles, then disappears from the vicinity of the target region in a time scale of <10–100 ns. Therefore, it is not clear whether related plasma effects, such as plasma reignition (absorption of impinging photons by the relaxing plasma), beam scattering, defocusing and prolonged plasma-target coupling, may have some influence on the micromachining process in terms of throughput and heat effect [32,33,34,35].

Though the above stated methods are applicable and are continuously being improved, it is difficult to produce a surface which would reflect the light in all directions, while maintaining its spectral components intact (intensity and color does not depend on the viewing angle). In this work, we propose a method by creating specific irregularities on the surface of the sample by means of laser ablation using bursts of femtosecond pulses. These irregularities need to meet specific conditions: (I) using a particular set of laser parameters, the laser-modified surface exhibits Lambertian or quasi-Lambertian light scattering properties that has a bright/white surface appearance. Moreover, (II) with a different set of laser parameters, the surface exhibits strong light absorbing properties caused due to nano-grating formation which gives a dark/black appearance. By combining the two regimes (black and white) it was also possible to create different shades of grey of the modified surface. The proposed method is suitable for creating a white color and gray shades on the surface of polished stainless-steel without the use of additional chemical or mechanical processes. The resulting white color and gray shades do not depend on the viewing angle, due to quasi-Lambertian scattering. By tailoring the properties of the surface, it is also possible to manufacture a non-Lambertian scattering surface, where the white color is visible only at certain range of angles, while in other angles a decrease of scattering efficiency and/or color tones may appear.

## 2. Materials and Methods

### 2.1. Experimental Setup for Ablation of Metal Sheets

**Figure 1:**
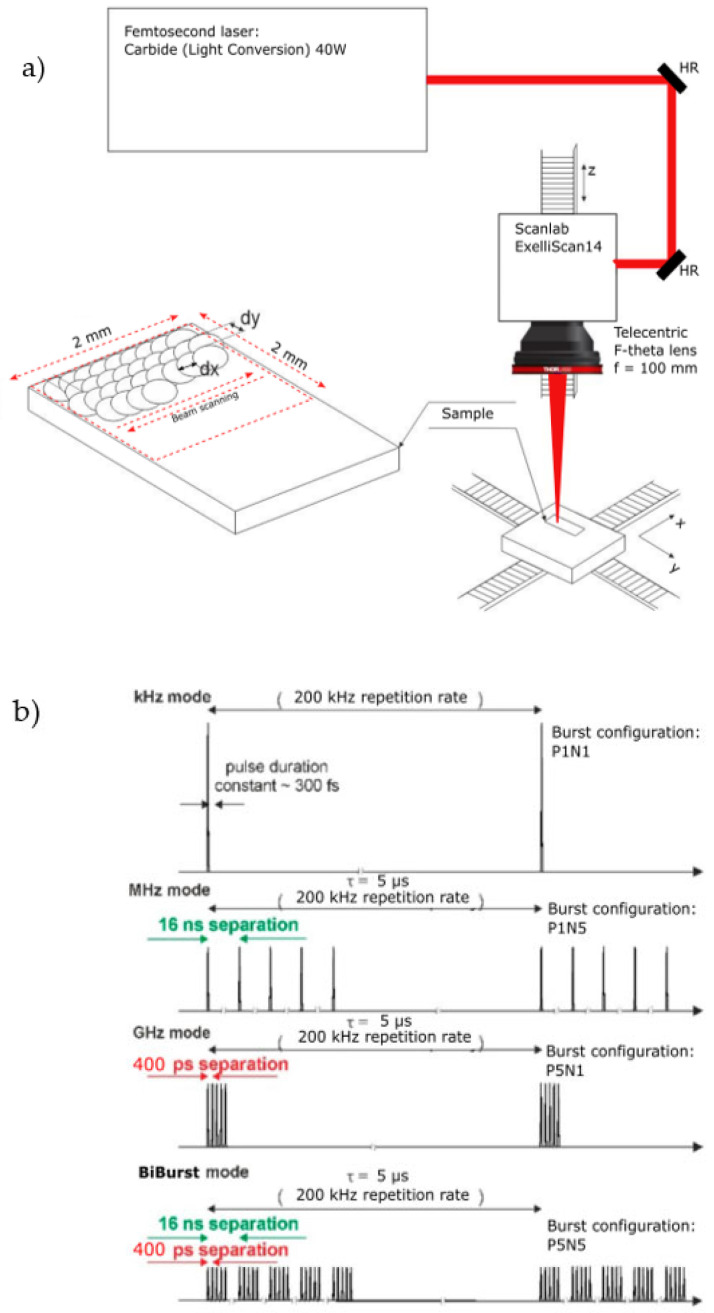
(**a**) Experimental setup for ablation of Stainless steel. The bottom part shows the fabrication algorithm used. (**b**) Illustration of the burst modes generated by the laser. The 16 ns temporal separation equates to a MHz, whereas the 400 ps to a GHz repetition rate. The BiBurst mode is a combination of the MHz and GHz modes and produces several pulses temporally separated by 400 ps, which are grouped by identical trains spaced by 16 ns. The integrated energy per cycle is the same for all the different burst modes.

A multi-burst (BiBurst) laser Carbide^®^ (Light Conversion Ltd., Vilnius, Lithuania) was used to produce bursts of femtosecond pulses temporally separated in the ~100 ps–10 ns time scales with a central wavelength of 1030 nm. The maximum average power of the laser was 40 W. Bursts of femtosecond pulses could be generated at a repetition rate ranging from 1 MHz to a single shot (however, in the experiments, the repetition rate was fixed to 200 kHz), and the bursts of pulses can be produced with separation times ranging from 400 ps to 16 ns. In addition, a combined packet of pulses separated by both 400 ps and 16 ns can also be realized, and this was called the BiBurst mode. The illustration of the bursts and its modes are depicted in Figure 1b. The formation of femtosecond bursts is done by injecting a specific number of laser pulses from the master oscillator into the regenerative amplifier at different instances in time and amplifying the injected pulses (burst) during multiple round-trips in the optical cavity of the regenerative amplifier and afterwards ejecting the amplified pulses (burst) from the cavity [36]. The energy of each pulse within the burst in the whole average power range was measured using the time-correlated single-photon counting method [37] and had a variation of no more than 10% standard deviation. Using a galvo-scanner (Excelliscan14, Scanlab Ltd., Puchheim, Germany) and a telecentric f = 100 mm F-theta lens, the pulses were focused on the sample. The beam was translated along the surface of the sample. The experimental setup is shown in Figure 1a. The beam waist was positioned directly on the surface of the sample. The spot size (diameter) at the focal position was measured with a CCD camera and was equal to 33 µm (1/e^2^). The Rayleigh length for this beam was calculated to be 0.9 mm. The investigated material was AISI304 stainless-steel sheets (1 mm thick). The dimensions of each sample were 5 cm × 5 cm. All samples were polished and had a mirror-like surface quality.

The experiments were carried out by scanning the beam along the surface of the sample multiple times (the scanning path was repeated up to 40 times and is denoted as N_P_) at different marking parameters which are listed in Table 1. For each parameter set 2 mm × 2 mm squares were fabricated. The spatial overlap dx, dy was varied from 9 to 70% by adjusting the scanning speed of the beam and the separation between adjacent lines (as shown in Figure 1a). The overlap was constant in the x and y directions, therefore dx is always equal to dy in all cases. The F/F_th_ denotes fluence ratio compared to the ablation threshold, which was verified experimentally to be 0.22 J/cm^2^. The burst configurations P denote the GHz-type burst (picosecond temporal separation of sub-pulses), N denotes the MHz-type burst (nanosecond temporal separation of sub-pulses), the PN configurations denote the combined regime or BiBurst mode (refer to Figure 1b). It should be noted, that when varying the burst configuration, the spatial overlap does differ slightly, i.e., if comparing the case of P1N1 to P1N8, the spatial separation is slightly different even if the scanning speed and the line overlap ar kept constant. However, since the temporal separation between the sub-pulses is <16 ns, the lowest separation between the sub-pulses within the burst (while the beam is being moved) is >99.7%, (for the GHz case, this value is ~99.99%); therefore, we deem that the variation in the spatial separation is negligible when varying the burst configurations. After micromachining, the brightness of the produced squares was evaluated using a Leica DM2500M microscope and an IDS CMOS camera using µEYE Cockpit software. For the evaluation of the reflected light from the exposed surface scattering angles, the 3D surface profile was measured using a Olympus LEXT OLS500 laser scanning microscope.

### 2.2. Theoretical Insights into the Formation of Lambertian Scattering White Surfaces

The white color/appearance can be generated by making a highly scattering, Lambertian-like surface that scatters all visible light at all angles equally. This is achieved by exposing the surface to laser radiation in such a way, that a rough surface of required dimensions and topology is created. A specific scattering of light can be generated by modifying the surface with bumps and dimples of specific diameter. The roughness of the surface that has the Lambertian-like scattering can be estimated by the Rayleigh roughness parameter *Ra_r_*. The surface is qualified as very rough if the parameter *Ra_r_* > 1 (according to [38]). If this condition *Ra_r_* > 1 is met for all the visible optical range, the surface will appear to an observer as white (if the material absorption is not accounted for). This parameter *Ra_r_* can be expressed by the following formula:

(1) $$Ra_{r}=k\sigma_{h}cos\left(\theta \right),$$

where k is the wavenumber, $\sigma_{h}$ is the root-mean-square height, and θ is wave incident angle. By defining θ equal to 0, the formula becomes $Ra_{r}=k\sigma_{h}$. If the lower limit of the visible spectrum is defined to be 740 nm and *Ra_r_* is equal to 1, then the surface must have $\sigma_{h}$ larger than 118 nm. Experimentally, the surface roughness can also be measured as an arithmetical mean deviation:

(2) $$Ra=\frac{1}{n}\left(\overset{n}{\underset{i=1}{\sum }}\left|z_{i}-\bar{z}\right|\right),$$

in this case, the correspondence of the parameter Ra to a very rough surface is not so straightforward. However, from the experimental evidence it can be estimated that geometrical magnitudes of the formed dimples/pits/vias/blind holes should meet the following conditions:

- Change in the height of the material due to manufactured irregularities should be larger than 150 nm, to induce scattering over the full angular spectrum;
- The distance between the formed dimples in the transverse coordinates (X and Y) should be smaller than 10 µm to manufacture a uniform scattering surface. The area between the dimples must have a low roughness <30 nm (*Ra*) to avoid a multiple reflection scenario which would increase light absorption on the surface.

To meet these surface roughness conditions, the surface roughness parameter Ra will be larger than 0.2 µm and the surface will become highly scattering. To achieve isotropic scattering, it is best to have a non-periodic, chaotic, or quasi-chaotic distribution of the roughness, because periodicity can be undesirable due to causing diffraction effects. The white-colored surface is achieved, when the machined surface is produced to be non-absorbent/reflective evenly over full optical range. In addition, given previous considerations it is possible with the same laser micro-machining setup to create regions which have an irregular sub-micron periodicity and produce uniform light-absorbing characteristics. In this case it is possible to regulate the shade of white of the surface. For example, by creation of a periodic sub-micron surface regions that have absorption in the visible optical range and simultaneously generating a Lambertian scattering surface it is possible to make a metal surface to appear grey. Using a single femtosecond pulse, it is typically not possible to initiate surface melting as ablation is the dominating effect, however, if 2 or more pulses within the burst are used, the melting temperature can be easily reached and controlled melting can be achieved [39].

## 3. Results and Discussion

**Figure 2:**
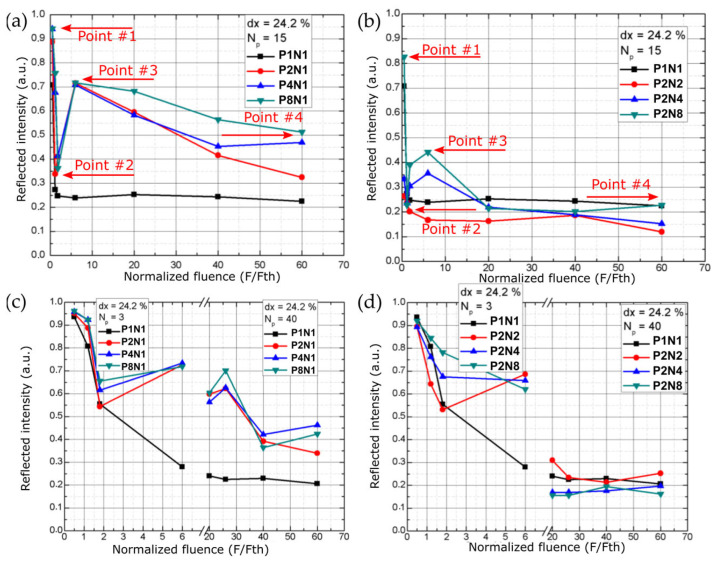
Brightness measurement results when scanning the 2 mm × 2 mm squares multiple times using different pulse energy settings and different burst mode configurations. dx denotes the spatial overlap of the pulses, Np denotes the number of scans (layers) on the surface. (**a**) Reflected intensity values as a function of the normalized fluence for different GHz burst modes. (**b**) Reflected intensity values as a function of the normalized fluence for different BiBurst modes. (**c**) Reflected intensity values as a function of the normalized fluence for different GHz burst modes when performing a different number of scans (layers). (**d**) Reflected intensity values as a function of the normalized fluence for different BiBurst modes when performing a different number of scans (layers).

**Figure 3:**
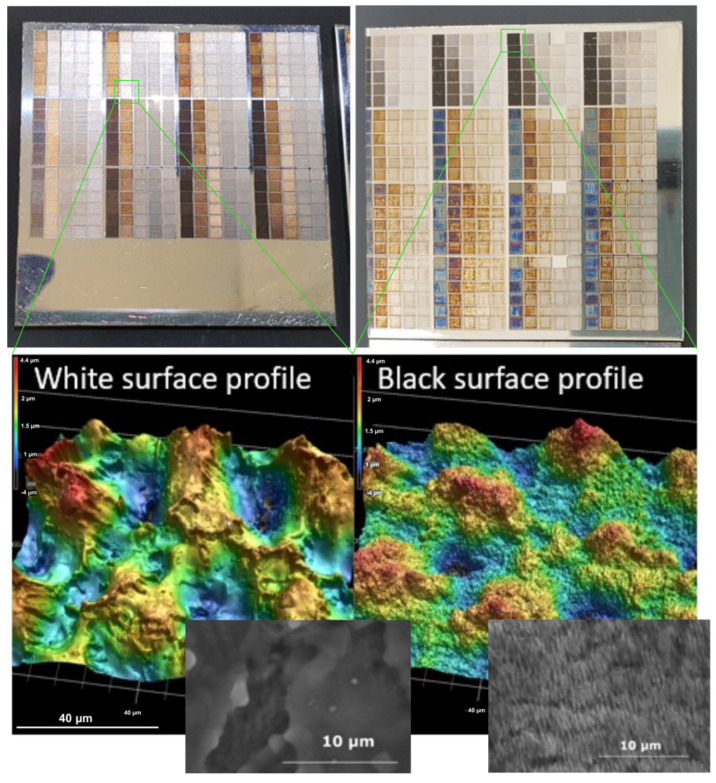
Micromachined surfaces images using different fabrication parameters (as listed in Table 1). The white markings reveal a required microstructure as discussed in Section 2.1, whereas the dark areas may exhibit a similar microstructure; however, with an embedded nanostructure which has light-absorbing properties.

**Figure 4:**
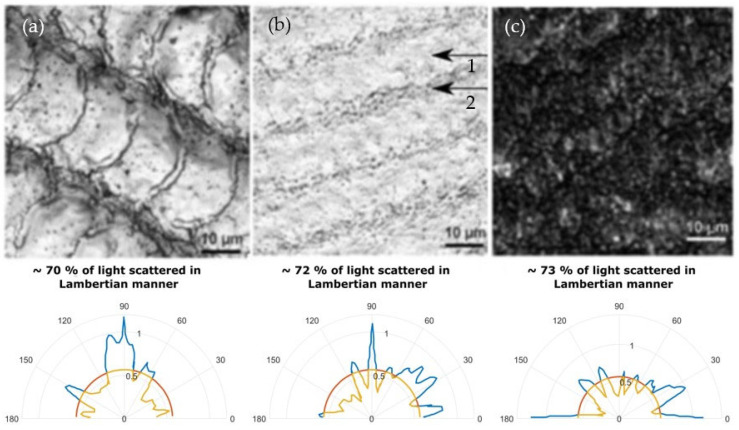
Laser microscope images of micromachined surfaces and their corresponding light scatter diagrams. (**a**)—represents white, (**b**)—gray and (**c**)—black color surface. Top row is SEM images, while at the bottom row is the scattering angle diagrams of these surfaces.

As per the listed parameter values in Table 1, the total amount of experimental points required to complete the full experiment equals 7425. Therefore, at the initial stage of experimentation, screening experiments were carried out to eliminate experimental points that are of no interest; in this case, this would be results that produce marked areas which do not exhibit a significant color change or the amount of reflected light is low. During screening, it was noticed that all of the experimental points done with the MHz burst mode (P1N2, P1N4, P1N8) do not produce adequate results and will be omitted from the analysis. After conducting surface analysis of the micromachined surfaces it was seen that the low reflectance from the surfaces produced within these regimes is caused by the multiple reflection scenario, i.e., even though it is possible to produce pits and dimples on the surface of the sample of required dimensions (described in Section 2.2), the areas between the dimples are covered with a nanostructure with poor light reflecting properties. Better results were achieved in terms of surface brightness when using the GHz mode or the combined BiBurst mode. Figure 2 shows the brightness measurement results when scanning the 2 mm × 2 mm squares multiple times using different pulse energy settings and different burst mode configurations. Several points are marked in Figure 2 in order to discuss the result further. Point #1 in Figure 2a and b shows the highest reflected intensity; however, in this case, the fluence is set below the ablation threshold in which case the surface exhibits negligible change. If the fluence is increased, regardless of the set spatial overlap of the pulses the brightness decreases up to point #2. In this case, the surface is being ablated and a rough surface is created which exhibits light absorbing (light trapping) structures. If the fluence is increased further, the reflected light intensity increases up to point #3. Upon inspection of the samples, it was found that the remelting of the surface starts to occur in the fluence region from point #2 to point #3. Point #3 shows the highest brightness of the surface. Upon inspection it was revealed that this is due to the low roughness within the exposed area which can reach values below 50 nm Ra, this may be seen as a gentle remelting of the top surface layer without undesired oxidation occurring (such as displayed in Figure 3); however, and inherent waviness of the surface may be present (and is mandatory according to the consideration described in Section 2.2). If the fluence is increased further from point #3 to point #4, the brightness starts decreasing. The decrease in brightness within this regime (from point #3 to point $4) is attributed to the overheating of the surface, in which case, self-organized microstructures may appear accompanied by oxide formation (which gives rise to a color change from white to brown or other colors). These tendencies (point #1 to point #4) were observed regardless of the used overlap or number of scans. If comparing plot a and b in Figure 2, when increasing the number of scans, the amount of reflected light does not drop as fast for the GHz case (Figure 2c) as it does for the BiBurst case (Figure 2d). Upon inspection it was noticed that for the Biburst case, the amount of removed material from the surface may be up to 10-fold higher as compared to the GHz case. This may be caused do the interaction of the sub-pulses within the burst with the plasma cloud that gets ejected from the surface, whereas in the Biburst case it is not as pronounced in comparison since the sub-pulses arrive later when the plasma cloud has dispersed. For the Biburst case, if the sample is scanned multiple times (>40) the amount of reflected light becomes similar to the conventional single-pulse case. In addition, the amount of reflected light from the surface is similar when comparing the GHz and Biburst cases after 3 scans (Figure 2c,d); however, after 15 scans (Figure 2a,b) the amount of total reflected light is lower in the BiBurst case (Figure 2b) as compared to the GHz case (Figure 2a). Since the amount of removed material is higher for the Biburst case as compared to the GHz case, a microstructure is formed in the BiBurst case, whereas the surface remains relatively flat for the GHz case. A few results and images of the micromachined samples are presented in Figure 3. It can be seen that some of the produced squares exhibit a white appearance (due to the above-mentioned conditions) whereas others range from black (light-absorbing nanostructure) to shades of gray. The gray shades may be explained as a combination of the white surfaces embedded with patches of nanostructured clusters, as is shown in Figure 4.

**Figure 5:**
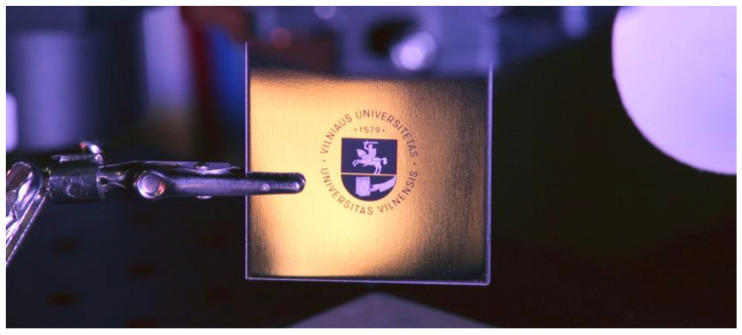
Vilnius University logo marked with the presented high-contrast marking method.

Taking into consideration the insights provided in Section 2.2, a microstructure is necessary to produce a surface which not only has low light absorption characteristics but also scatters light in a Lambertian manner. To be able to investigate the scattering properties of the surface, 3D topographies of the micromachined areas were measured with a laser scanning microscope. We have used the height difference within the microstructure to modify the phase of the impinging and reflected plane wave from the surface. By taking the Fourier transform of the modified wave it is possible to deduce the scattering angles of light. Such a result is displayed in Figure 4a, in Figure 4 it shows a white surface, similar to one displayed in Figure 3. In this case, it was found that > 70% of the reflected light is scattered in a Lambertian manner. In which case, the surface appears white not only perpendicular to the viewing direction but also at different angles. Image b) shows a shade of white or a gray color. The surface has a grey appearance since some areas within the melted surface (arrow #1) exhibit nanostructured patches (arrow #2). Because of this, the even though the light is reflected in a Lambertian manner, a portion of the light is absorbed within these regions thus producing a gray appearance. The dark surfaces in c) also exhibit Lambertian-like light scattering characteristics. In this case, there are no melted areas and the whole surface is covered with irregular nanostructures. The irregularity is beneficial to maintain the dark appearance at all angles as the surface does not act as a diffraction grating and does not scatter different wavelength components at different angles. It is worth mentioning that even though the experiments were carried out on stainless-steel samples, if the above-mentioned conditions for light scattering are met, the same color and scattering characteristics may be achieved on other materials, such as different metallic materials, dielectrics, and semiconductors. However, the color appearance is dependent on material properties, i.e., if the reflection spectrum in the visible region is uniform, then the surface may appear white. However, if the spectrum is not uniform, such as for the case of gold, then a different appearance may emerge since certain wavelength components are not reflected or are attenuated. What is more, if the illumination source is not white, then the appearance of the white-marked areas may also differ, as is shown in Figure 5, where the marked sample is illuminated with a cold spectrum LED lamp (with more expressed blue/violet spectral components).

The extensive experimental analysis shows that the white-appearance surfaces can be achieved with multiple parameter conditions as long as at least two or more either GHz-mode or BiBurst-mode sub-pulses are used, whereas the gray and black surfaces can be achieved using the MHz mode as well. The highest brightness (lowest absorption from the surface) is typically realized when the fluence setting is above the ablation threshold for a single pulse (non-burst mode) by 5–10-fold. As an example of this type of high-contrast marking, the Vilnius University logo was marked with black and white colors and is displayed in Figure 5. For the white-marking the burst configuration of P2N8 was used, the overlap was set to 24%, the laser was operating at a repetition rate of 200 kHz, the fluence was set to 1.6 J/cm^2^ and the whole pattern was repeated 15 times. For the dark-marking the burst configuration of P1N2 was used, the overlap was set to 70%, the laser was operating at a repetition rate of 200 kHz, the fluence was set to 5 J/cm^2^, and the whole pattern was repeated 15 times.

## 4. Conclusions

We have presented the results of high-contrast marking of stainless-steel (AISI304) samples with white, gray, and black colors. Different burst modes were investigated that equate to GHz, MHz, and kHz repetition rates, as well as a combined GHz and MHz mode- Biburst. It was found that if the fluence is set to approximately 5–10-fold the ablation threshold of the single-pulse case and at least two sub-pulses within the burst are used in the GHz and Biburst modes, it is possible to create a white surface which scatters light in a quasi-Lambertian manner granted that the Rayleigh scattering conditions are met. The white appearance is a result of a specific laser produced surface morphology, i.e., a microstructured, rough remelted layer on the surface, which exhibits poor light-absorbing properties. It was also shown that the gray and black colors on the surface originate due to irregular nanostructure formations which have light absorbing (light trapping) characteristics. The control between micro and nano structure density, and consequently surface scattering and absorption parameters, can be achieved by changing the laser burst pulse parameters.

## Figures and Tables

**Table 1:** Varied parameter settings in the experiments.

Parameter	Setting, Value
**F/F_th_**	0.5, 0.7, 1.2, 1.8, 3, 4, 6, 14, 20, 26, 32, 40, 46, 53, 60
**Scanning overlap dx, dy (%)**	9, 24, 39, 54, 70
**# of repetitions (N_P_)**	1, 3, 6, 9, 12, 15, 20, 25, 30, 35, 40
**Burst configuration**	P2N1, P4N1, P8N1, P2N2, P2N4, P2N8, P1N2, P1N4, P1N8
**Wavelength (nm)**	1030
**Laser rep. rate (kHz)**	200

## Data Availability

The data presented in this study is available on request from the corresponding author.
